# Use of Liquid Platelet-Rich Fibrin (Liquid PRF) as a New, Effective Regenerative Treatment for Steroid-Induced Subcutaneous and Dermal Atrophy

**DOI:** 10.7759/cureus.109245

**Published:** 2026-05-19

**Authors:** Sima Aalami Harandi, Sara Saadat, Richard J Miron, Nima Farshidfar, Kamran Balighi

**Affiliations:** 1 Department of Dermatology, Parsian Laser Clinic, Bandar Abbas, IRN; 2 Department of Anesthesia and Pain Medicine, Iran University of Medical Sciences, Tehran, IRN; 3 Department of Periodontology, University of Bern, Bern, CHE; 4 Department of Dermatology, Tehran University of Medical Sciences, Tehran, IRN

**Keywords:** dermal atrophy, liquid prf, platelet-rich fibrin, prf, skin atrophy, soft tissue regeneration, steroid atrophy, subcutaneous atrophy

## Abstract

Subcutaneous and dermal atrophy are known complications of corticosteroid injections. Due to aesthetic and/or functional concerns, such as fat loss, visible depressions, skin thinning, and discoloration of the affected area, an effective treatment modality for reconstructing the area remains needed. Considering the regenerative properties of platelet-rich fibrin (PRF), its potential in soft tissue repair, and its efficacy in aesthetics, we used this natural autologous biomaterial to treat steroid-induced dermal and subcutaneous atrophy in our patient.

A 35-year-old woman developed a well-defined atrophic area measuring approximately 3 × 7 cm² in the scapular region following a triamcinolone injection for shoulder pain. The area was characterized by a pronounced depression with irregular indented borders, along with dermal thinning and dyspigmentation. Treatment included three sessions of liquid PRF injections at monthly intervals. Each PRF injection session resulted in progressive improvement in the depth and quality of the atrophic area. One month after the third session and at six-month follow-up, significant improvement with marked restoration was observed in subcutaneous tissue volume, skin texture, depigmentation, and overall aesthetic outcomes based on subjective clinical assessment. No adverse effects were observed during treatment and follow-ups.

Liquid PRF may represent a promising regenerative treatment for steroid-induced subcutaneous and dermal atrophy, warranting further controlled studies. Its potential to improve the thickness, volume, texture, and quality of both the dermal and subcutaneous fat layers may support the role of PRF as a therapeutic option for soft tissue restoration in dermatology and aesthetic medicine.

## Introduction

Corticosteroid injections, due to their wide therapeutic beneficial effects, are commonly used in various medical fields, including dermatology, internal medicine, orthopedics, and pain management. However, an important and undesirable complication of steroid injections is steroid-induced subcutaneous and dermal atrophy. The severity and size of such skin atrophy are influenced by several important factors, including the type, concentration, amount, depth, injection site, and the technique of steroid injection [1,2]. Skin manifestations of steroid-induced atrophy include a reduction in dermal thickness and elasticity, changes in skin texture and color, and visible depressions or indentations due to reduced subcutaneous thickness and fat loss. Such atrophies can significantly impact patients’ aesthetic and functional concerns [3]. The pathophysiology underlying steroid-induced atrophy includes inhibition of fibroblast proliferation, decreased synthesis of collagen types I and III, lipocyte apoptosis with fat involution, and vascular compromise [1-3].

For the treatment of steroid-induced dermal and subcutaneous atrophy, various modalities, including hyaluronic acid fillers [4], fat grafting [5], platelet-rich plasma (PRP) [6], fractional CO_2_ lasers, and surgical approaches have been introduced. However, these treatments have limitations such as variable outcomes, technical complexity, cost, and, more importantly, limited regenerative capacity at both dermal and subcutaneous tissues. Therefore, an optimal regenerative solution remains a clinical need.

Among the emerging regenerative options, platelet-rich fibrin (PRF) has recently gained attention due to its promising biological properties. PRF is a natural autologous biomaterial with demonstrated efficacy in soft tissue regeneration, wound healing, and aesthetic medicine [7-9]. There are two types of PRF based on centrifugation protocols and clinical needs: solid and liquid (injectable) forms. PRF is prepared through a simple blood centrifugation process, resulting in a fibrin matrix rich in platelets and leukocytes [7-10]. The slow and sustained release of various growth factors and cytokines over 10-14 days at the utilized area enables PRF to function as a bio-stimulant, promoting robust tissue regeneration in both dermal and subcutaneous layers [8,9,11-13]. Various studies have shown that PRF can support volume restoration in subcutaneous tissues and enhance skin texture, thickness, and overall quality by stimulating angiogenesis, fibroblast proliferation, and the production of collagen and extracellular matrix (ECM) components [7,8,14-16].

Moreover, compared to PRP, liquid PRF is a completely natural biomaterial without additives or anticoagulants, requires a simpler and shorter preparation process, and provides a more gradual and prolonged release of growth factors and cytokines due to its three-dimensional fibrin scaffold, resulting in enhanced regenerative potential [8,9,12,17].

Considering this scientific background and our prior positive clinical experience in employing liquid PRF in the aesthetic and dermatology field, we decided to treat a depressed, steroid-induced skin atrophy with liquid PRF. To the best of the authors’ knowledge, it seems to be the first case report documenting the use of PRF for the treatment of steroid-induced subcutaneous and dermal atrophy.

## Case presentation

A 35-year-old woman with Fitzpatrick skin type III presented with skin atrophy (approximately 3 × 7 cm) on the upper back (scapular region) following a triamcinolone injection for shoulder pain approximately one month earlier. The affected area was severely depressed, with obvious irregular, indented borders and marked dyspigmentation, including areas of depigmentation, hypopigmentation, and hyperpigmentation.

After obtaining written informed consent, three liquid PRF injection sessions were performed at one-month intervals. In each session, liquid PRF was prepared using two plastic blood collection tubes and a fixed-angle centrifuge device (IntraSpin, Germany) at 2,700 rpm for three minutes (maximum relative centrifugal force (RCF) ≈ 700 g). Each tube yielded approximately 3.5 mL of liquid PRF. The injections were administered using a 27-G, 19-mm needle through multiple injection points, primarily in the subcutaneous layer and to a lesser extent in the dermis, including the indented borders of the atrophic area.

In the first session, approximately 7 mL of PRF was injected. This covered nearly the entire depressed, lipoatrophic area, resulting in it becoming almost level with the surrounding skin. In the second session, considering the obvious improvement in overall appearance and the depth of the atrophic area (≈60% improvement based on clinical and photographic estimates), we injected 5 mL of PRF, mostly into the subcutaneous fat and less into the dermis. In the third session, due to prominent improvement in the depth of the depressed area, as well as improvement in skin texture, quality, and color, approximately 3 mL of PRF was injected into the remaining affected zones. Patient-reported pain during the injection sessions was mild (numeric rating scale (NRS): 2/10) [18]. No specific post-procedure care was required.

At one-month follow-up after the final session, the treated area was no longer visibly depressed, and dermal atrophy was no longer evident. Only some hyperpigmentation, as observed initially, remained. A follow-up visit at six months clearly demonstrated the same promising improvement in the atrophic area, with even better skin quality.

**Figure 1:**
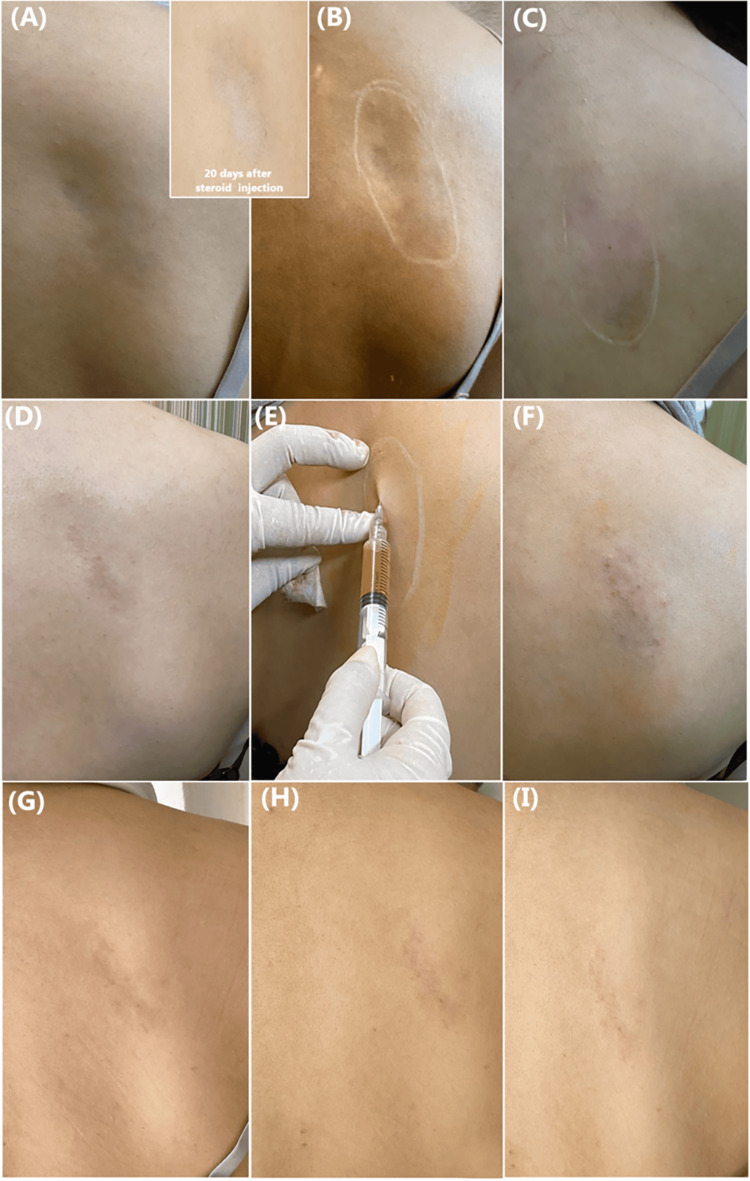
Sequential clinical improvement after three sessions of liquid PRF injections for steroid-induced atrophy. (A) Before treatment: one month after steroid injection in the upper back (scapular region), demonstrating cutaneous atrophy; (B) marking of the atrophic area; (C) immediately after the first PRF injection, primarily into the subcutaneous tissue; (D) one month after the first PRF injection; (E) second PRF injection session; (F) immediately after PRF injection into the subcutaneous and dermal layers; (G) one month after the second injection; (H) one month after the third injection session; (I) six-month follow-up demonstrating remarkable improvement of the depressed area and dermal atrophy.

At both one- and six-month follow-ups, the patient reported “very much improved” on the Patient Global Impression of Change (PGIC) scale [19], indicating high satisfaction with the aesthetic outcome. Clinical assessment using the Physician Global Aesthetic Improvement Scale (GAIS) [20] rated the result as “much improved,” reflecting significant restoration of tissue volume and skin texture. No adverse effects were observed during treatment or follow-ups. Figure 1 shows the initial atrophic area and the progressive clinical improvement following serial monthly liquid PRF injections.

## Discussion

This case report presented liquid PRF as an effective regenerative approach for treating steroid-induced subcutaneous and dermal atrophy, a common and challenging complication of steroid injections. The findings demonstrated remarkable improvements in tissue thickness, skin texture, elasticity, color, especially depigmentation, and nearly complete correction of a 3 × 7 cm² atrophic depression. These improvements may be attributed to the well-established potency of PRF for soft tissue regeneration.

As an autologous blood-derived biomaterial, PRF is rich in platelets, leukocytes, growth factors, cytokines, and creates a three-dimensional fibrin matrix scaffold, which are key components of tissue regeneration, healing, and repair [8,9,13,15]. The rich content of growth factors in PRF, such as vascular endothelial growth factor (VEGF), transforming growth factor beta (TGF-β), and platelet-derived growth factor (PDGF), promotes angiogenesis, regulates cell proliferation, and supports the migration and differentiation of cells into fibroblasts. Additionally, PRF has been shown to enhance fibroblast activity, collagen synthesis, and ECM deposition, contributing to effective tissue regeneration [8,13,15]. In the fat layer, PRF has been shown to enhance fat cell viability, prevent apoptosis, and promote adipogenesis [14,15].

The mechanisms described above for promoting tissue regeneration and enhancement are precisely those required to effectively treat steroid-induced skin atrophy. Indeed, tissue regeneration with PRF, referred to as natural guided regenerative therapy, mimics the wound healing process [16], the body’s natural, potent, and efficient mechanism for repairing tissue injuries or defects, even in large wounds.

This new treatment offered considerable advantages, including the use of an autologous, biocompatible, and cost-effective regenerative biomaterial, demonstrating restorative potential for both dermal and subcutaneous tissue. Moreover, the preparation method is simple, quick, and does not require any anticoagulants or additives. PRF has also been used successfully in numerous dental and medical fields for over 20 years [9,10,13,15], and its outcomes have been reported to be superior to those with PRP [11,17]. These combined factors were the rationale for selecting PRF as a potential treatment option in our study.

Our results were also compatible with the findings of other studies in the field of aesthetic medicine and dermatology [8-10,21]. In rejuvenation, PRF has been injected both in dermis and subdermal fat for improvement of skin wrinkles, thickness, elasticity, and also volumization as a filler [10,21]. Additionally, Ardekani et al. have shown that subdermal injection of PRF for correction of the nasolabial fold has led to a significant increase in subcutaneous fat thickness, as proven by sonography after three months [21]. PRF has also been used successfully for dermal reconstruction in striae [8] and acne scars [11]. Moreover, PRF has been used in the treatment of vitiligo and melasma [8], demonstrating its potential to improve skin pigmentation, as observed in our case with the correction of depigmentation. Lastly, the use of solid PRF for refractory chronic leg ulcers, including large and deep ulcers, as well as sinus tracts resulting from postoperative wound dehiscence, represents another valuable application that underscores the effectiveness of PRF in soft tissue regeneration [12,16,22].

This case study presented liquid PRF as an effective, easy-to-apply, safe, and minimally invasive regenerative approach for the treatment of steroid-induced cutaneous atrophy. For achieving the best outcome with this new treatment, the following points are noteworthy: (1) using an appropriate centrifuge and employing accurate centrifugation protocols, including speed (rpm), RCF (g), and time for preparing PRF. Since it has been shown that horizontal centrifugation can provide a more uniform cell distribution and higher cell concentration, using such a centrifuge might lead to even better outcomes [9,23]; (2) marking the atrophic area and injecting sufficient amounts of PRF into the whole area; and (3) repeating the treatment every four weeks. Considering the limitations of this study, including a single case and subjective clinical assessment, further studies using objective assessment tools such as ultrasound will provide more validation for the use of PRF in treating steroid-induced skin atrophy.

## Conclusions

Liquid PRF seems to be a safe, effective, and scientifically grounded regenerative modality for treating steroid-induced cutaneous atrophy. By stimulating angiogenesis, fibroblast proliferation, ECM synthesis, collagen remodeling, and adipogenesis, PRF directly counteracts the underlying tissue deficits through durable regeneration of both dermal and subcutaneous tissues. Its regenerative capacity may also support broader applications across dermatology and aesthetic medicine.
